# Bi-directional regulation of cartilage metabolism by inhibiting BET proteins—analysis of the effect of I-BET151 on human chondrocytes and murine joints

**DOI:** 10.1186/s13018-018-0797-y

**Published:** 2018-05-21

**Authors:** Jin Dai, Sheng Zhou, Qiting Ge, Jinzhong Qin, Dongyang Chen, Zhihong Xu, Dongquan Shi, Jianxin Li, Huangxian Ju, Yi Cao, Minghao Zheng, Chao Jun Li, Xiang Gao, Huajian Teng, Qing Jiang

**Affiliations:** 1 Department of Sports Medicine and Adult Reconstructive Surgery, Drum Tower Hospital, School of Medicine, Nanjing University, 321 Zhongshan Road, Nanjing, 210008 Jiangsu People’s Republic of China; 2 Model Animal Research Center of Nanjing University, Xuefu Road, Nanjing, 210032 Jiangsu People’s Republic of China; 3 State Key Laboratory of Analytical Chemistry for Life Science, Nanjing University, Hankou Road, Nanjing, 210093 People’s Republic of China; 4 Collaborative Innovation Center of Advanced Microstructures, National Laboratory of Solid State Microstructure and Department of Physics, Nanjing University, Hankou Road, Nanjing, 210093 People’s Republic of China; 5 Sir Charles Gairdner Hospital, School of Surgery, The University of Western Australia, 35 Stirling Highway, Perth, 6009 Australia; 6 State Key Laboratory of Pharmaceutical Biotechnology and Jiangsu Key Laboratory of Molecular Medicine, Model Animal Research Center and School of Medicine, Nanjing University, Nanjing, 210093 People’s Republic of China

**Keywords:** Osteoarthritis, Chondrodyte, Matrix-degrading enzymes, Cartilage anabolism, Brd

## Abstract

**Background:**

Proinflammatory cytokines, which can upregulate the expression of matrix-degrading enzymes in chondrocytes, play important roles in the development of osteoarthritis. And a BET protein inhibitor, I-BET151, has been shown to exert an anti-inflammatory effect by repressing the BET protein-mediated expression of inflammatory genes. Our objective is to investigate the effect of I-BET151 on a surgical mouse model of osteoarthritis (OA) and human chondrocytes.

**Methods:**

We first treated a surgical mouse model of OA with I-BET151 once per day and evaluated the knee joints at 6 and 8 weeks after treatment. We then pretreated the human chondrocytes with I-BET151 prior to treatment with IL-1β or TNF-α and checked the expression and activity of the matrix-degrading enzyme genes. We also checked the expression of *ACAN*, *COL2A1*, and *SOX9*.

**Results:**

We demonstrated that I-BET151 could prevent articular cartilage damage in the surgical mouse model of OA at an earlier time after treatment, but not at a later time after treatment. I-BET151 could robustly suppress the IL-1β- and TNF-α-induced expression and activity of several matrix-degrading enzymes in human chondrocytes. I-BET151 could also suppress the expression of *ACAN*, *COL2A1*, and *SOX9*.

**Conclusions:**

Our findings suggested that inhibiting BET proteins could exert a repression effect on both of chondrocyte anabolism and catabolism, and the effect of BET protein inhibitor on surgical mouse model of OA needs further evaluation.

## Background

Osteoarthritis (OA) is the most common multifactorial disorder of the joints and is mainly characterized by the progressive degeneration of articular cartilage. It has been well documented that proinflammatory cytokines play important roles in the onset and progression of OA [1–3]. Among these cytokines, interleukin (IL)-1β and tumor necrosis factor (TNF)-α can upregulate the expression of several matrix-degrading enzymes. These matrix-degrading enzymes include matrix metalloproteinase (MMP) 1, MMP3, and MMP13 and a disintegrin-like and metalloproteinase with thrombospondin type 1 motifs (ADAMTS) 4 and ADAMTS5, which are involved in cartilage degradation in OA [1]. However, because of the complexity of IL-1β and TNF-α regulation, targeted therapy against these proinflammatory cytokines in OA is far from satisfactory [4].

In mammals, the bromo and extra-terminal (BET) family of proteins consists of ubiquitously expressed Brd2, Brd3, Brd4, and testes/oocyte-specific Brdt [5, 6]. Small molecular inhibitors that block BET proteins from recognizing acetylated histones have been generated recently [7]. BET protein inhibitors show therapeutic efficacy in cancer and inflammation diseases [8–12]. Recent reports revealed that I-BET151, a BET family protein inhibitor, suppressed the expression of inflammatory genes induced by IL-1β and TNF-α in rheumatoid arthritis synovial fibroblasts and inhibiting BET proteins can ameliorate K/BXN serum-induced arthritis [13, 14], while another study suggested that inhibiting BET proteins can suppress chondrocyte differentiation [15]. There have been reports that inhibiting the inflammatory response induced by IL-1β and TNF-α had protecting effect on OA progress in surgical mouse model of OA [16, 17], and anti-inflammatory drugs, such as COX-2 inhibitor and diacerein, can be the common treatment for both rheumatoid arthritis and OA [18, 19]. But considering the general inhibiting effect of BET inhibitors, the effect of BET inhibitors on OA may be different from other anti-inflammatory reagents. So, we wonder whether BET inhibitors can be effective for treating OA.

In the present study, we examined the effect of I-BET151 on a surgical mouse model of OA. Then, we examined the effect of I-BET151 on regulation of matrix-degrading enzymes in human chondrocytes. We also examined the effect of I-BET151 on regulation of cartilage matrix genes in human chondrocytes.

## Methods

### Animal and reagents

Male 129 S1/SvImJ mice (8 weeks) were obtained from the Model Animal Research Center of Nanjing University. All animal experimental procedures were conducted in compliance with institutional guidelines and approved by the Institutional Animal Care and Use Committee of Nanjing University. Animals were maintained on standard rodent chow and had free access to food and water. All reagents were purchased from Sigma unless otherwise indicated. Dimethyl sulfoxide (DMSO) was used as the vehicle for I-BET151, IL-1β, and TNF-α.

### OA model and histological analysis

The surgical procedures for destabilization of the medial meniscus (DMM) surgical models in the mouse have been described previously [20]. In this study, each group had 10 animals. The operations were performed under a surgical microscope. DMM surgical models were created through transecting medial meniscotibial ligament. Sham operations were identical except that the ligament was not transected. Sham operations were performed on independent mouse. All the operations were performed when the animals were at age of 10 weeks. To assess therapeutic effect of I-BET151, either I-BET151 (10 mg/kg) or DMSO was administered by intraperitoneal injection (once per day) for test or control group from 2 days after the operation to 1 day before sacrifice. At 6 and 8 weeks after treatment, the mice were sacrificed. All the mice survived until being sacrificed. The knee joints were fixed in 4% paraformaldehyde for 24 h and decalcified in EDTA for 1 week. The samples then were embedded in paraffin and successive sections 5 μm thick were prepared. Sections were stained with safranin O/fast green. Histological assessment of OA was quantified by OARSI histopathology grading [21]. All the sections of each studied joint were examined and assessed independently by two blinded investigators (JD and SZ), and the score for the most severe section of each joint was recorded. A third investigator (QG) exchanged the most severe sections selected by the two blinded investigators to each other if different sections were selected for one joint, and the deriving information of each section was blind to the two investigators. The highest score for each joint from each investigator was recorded by the third investigator, and the average of two highest scores for each joint was used for analysis.

### Cell culture and treatment

Human samples were obtained with informed consent from the donors. The study was approved by the ethical committee of Drum Tower Hospital, Medical School, Nanjing University. Fresh cartilage samples (from three patients undergoing knee replacement surgery at Drum Tower Hospital, Medical School, Nanjing University) were chopped from the lateral condyle of the operated knee. All the three patients were males and suffered with OA. The age of the patients ranges from 64 to 68 years, and none of them has a history of immunological diseases. Primary human articular chondrocytes were cultured as previously described [22]. The cells were passaged at a 1:2 dilution, and the second to the fourth passages were used for assays.

For the cellular assays, the cells were grown to approximately 80% confluence and then starved (in medium containing 0.1% FBS) for 12 h. I-BET151 (1 μM; TOCRIS, R&D Systems) or DMSO was added 1 h prior to treatment with IL-1β (10 ng/ml; R&D Systems) or TNF-α (10 ng/ml; R&D Systems). The dose of I-BET151 was applied in the recent report about the effect of I-BET151 on rheumatoid arthritis synovial fibroblasts, and in the same report, the authors found that delayed treatment with I-BET151 showed lower effect than that pretreatment with I-BET151 [14]. The dose of IL-1β and TNF-α was mentioned in the previous studies of chondrocytes [23, 24].

### Gene transcript analysis

The total RNA from primary human chondrocytes was isolated using TRIzol reagent (Ambion, Invitrogen) after 24 h of treatment. First-strand cDNA was prepared by reverse transcription using the PrimeScript RT Reagent Kit according to the manufacturer’s manual (TaKaRa). Real-time PCR was performed in an ABI StepOnePlus instrument (Applied Biosystems) using SYBR Green PCR Master Mix (Thermo Scientific). The expression of glyceraldehyde-3-phosphate dehydrogenase (*GAPDH*) of each sample was set as 1, and the relative expression of other genes was calculated and recorded. The primers used in this study are listed in Table 1.

**Table 1:** The list of primers used in this study

Gene	Strand	Primer sequences (5′ to 3′)	Detection
MMP1	Forward	CTCTGGAGTAATGTCACACCTCT	RT-PCR
	Reverse	TGTTGGTCCACCTTTCATCTTC	
MMP3	Forward	AGTCTTCCAATCCTACTGTTGCT	RT-PCR
	Reverse	TCCCCGTCACCTCCAATCC	
MMP13	Forward	ACTGAGAGGCTCCGAGAAATG	RT-PCR
	Reverse	GAACCCCGCATCTTGGCTT	
ADAMTS4	Forward	GAGGAGGAGATCGTGTTTCCA	RT-PCR
	Reverse	CCAGCTCTAGTAGCAGCGTC	
MMP2	Forward	TACAGGATCATTGGCTACACACC	RT-PCR
	Reverse	GGTCACATCGCTCCAGACT	
MMP9	Forward	TGTACCGCTATGGTTACACTCG	RT-PCR
	Reverse	GGCAGGGACAGTTGCTTCT	
ADAMTS5	Forward	GAACATCGACCAACTCTACTCCG	RT-PCR
	Reverse	CAATGCCCACCGAACCATCT	
COL2A1	Forward	TGGACGATCAGGCGAAACC	RT-PCR
	Reverse	GCTGCGGATGCTCTCAATCT	
ACAN	Forward	CCCCTGCTATTTCATCGACCC	RT-PCR
	Reverse	GACACACGGCTCCACTTGAT	
SOX9	Forward	AGCGAACGCACATCAAGAC	RT-PCR
	Reverse	CTGTAGGCGATCTGTTGGGG	

### Protein expression analysis

Western blot assays were carried out as previously described [25]. The protein from primary human chondrocytes was isolated after 24 h of treatment. The primary antibodies used were rabbit polyclonal anti-MMP1 (1:500), anti-MMP2 (1:500), anti-MMP13 (1:500), anti-glyceraldehyde-3-phosphate dehydrogenase (GAPDH) (1:1000), goat polyclonal anti-MMP3 (1:500), mouse monoclonal anti-MMP9 (1:1000; Santa Cruz Biotechnology, Inc.), and anti-SRY-related high mobility group-box gene9 (SOX9) (1:2000; Millipore).

### Matrix-degrading enzyme activity assay

#### Gelatin zymography

The supernatants of primary human chondrocyte cultures were collected after 24 h of treatment and concentrated using an Amicon Ultra 10K device (Amicon, Millipore). The samples were separated with 10% SDS gels containing 0.1% (*W*/*V*) gelatin. After electrophoresis, the SDS was removed by washing the gels three times with renaturing buffer (2.5% Triton X-100) for 30 min at room temperature. The gels were subsequently incubated in zymogen development buffer (50 mM Tris-HCl, pH 7.8, 200 mM NaCl, 5 mM CaCl_2_, 0.02% Brij 35) at 37 °C for 20 h. After briefly washing in water, the gels were stained with Coomassie blue R-250 for 1 h and destained with 40% methanol and 5% acetic acid until clear sharp bands appeared over the background.

#### ADAMTS activity assay

The supernatants of primary human chondrocyte cultures were treated by the same way as above. ADAMTS activity was analyzed using a commercially available aggrecanase activity assay kit (Abnova), following the manufacturer’s protocol. The results were normalized to the protein concentration of whole cells.

### Statistical analysis

All data were expressed as the means S.E. and represent at least three independent experiments. Statistical comparisons were made using Student’s *t* test or one-way ANOVA with post hoc Tukey test. *P* < 0.05 was considered statistically significant. As the increase of *MMP1, MMP3*, *MMP13*, and *ADAMTS4* mRNA expression by stimulation of IL-1β and TNF-α was high and variable, we used log_2_ of relative expression of these four genes for data presentation and statistical analysis.

## Results

### Inconsistent results are observed at different time after treatment in a surgical mouse model of OA

**Fig. 1:**
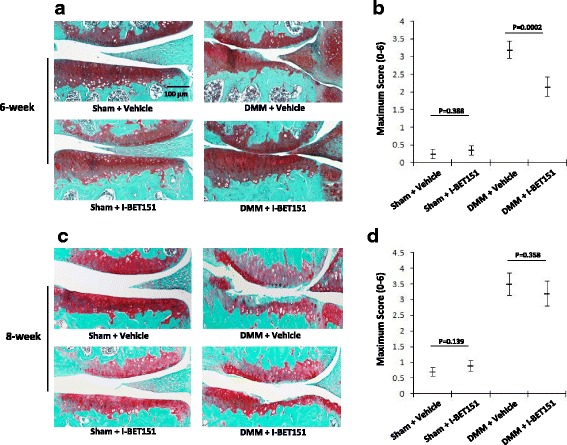
The effect of BET protein inhibitor on the severity of OA in a surgical mouse model of OA. **a** Representative Safranin-O-stained sections of the knees from test and control group, 6 weeks after surgery DMM. **b** Histological assessments of OA on femoral and tibial cartilage by OARSI histopathology grading, 6 weeks after surgery DMM (*n* = 10). **c** Representative Safranin-O-stained sections of knees from test and control group, 8 weeks after surgery DMM. **d** Histological assessments of OA on femoral and tibial cartilage by OARSI histopathology grading, 8 weeks after surgery DMM (*n* = 10)

In order to check the effect of I-BET151 on OA in vivo, we examined whether I-BET151 could exert an effect on protecting articular cartilage from degeneration during OA development in a surgical mouse model of OA. I-BET151 or vehicle was administered by intraperitoneal injection for DMM and sham group, respectively. After surgery for 6 weeks, an apparent damage of articular cartilage was observed in DMM groups comparing with sham groups. As expected, I-BET151 significantly alleviated the extent of damage of articular cartilage in the DMM groups (Fig. 1a, b). After surgery for 8 weeks, an apparent damage of articular cartilage was observed in DMM groups, but in both the DMM groups and the sham groups, the extent of damage of articular cartilage showed no significant difference between I-BET151-treated group and control group (Fig. 1c, d).

### The expression of IL-1β or TNF-α-induced matrix-degrading enzymes in chondrocytes is suppressed by BET protein inhibitor

**Fig. 2:**
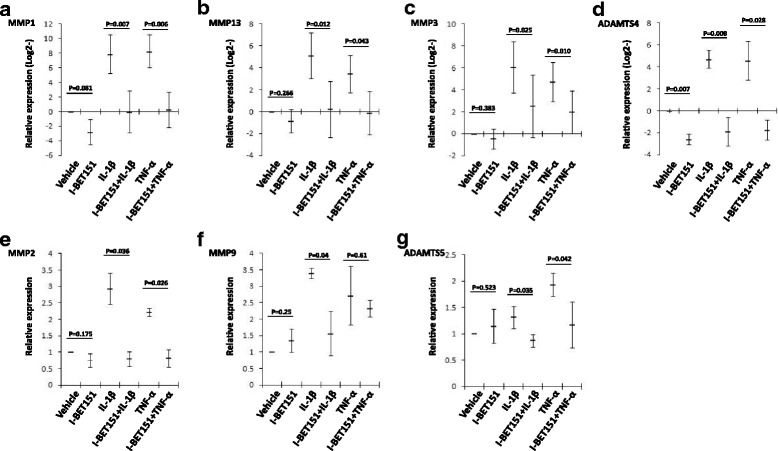
The effect of BET protein inhibitor on regulation of matrix degradation enzyme genes transcription in human chondrocytes (**a**–**g**). The transcriptional expression (RT-PCR) of *MMP1*, *MMP3*, *MMP13*, *ADAMTS4*, *MMP2*, *MMP9*, and *ADAMTS5* genes in human chondrocytes, respectively, after the cells were pre-treated with or without I-BET151 (1 μM) followed by addition of IL-1β (10 ng/ml) or TNF-α (10 ng/ml) for 24 h. Relative fold change values were calculated in comparison with vehicle control set as 1 (*n* = 3)

Since it was believed that IL-1β- and TNF-α-induced upregulation of matrix-degrading enzymes in articular chondrocytes played critical roles in OA development [1–3], we therefore investigated the roles of BET family proteins in the transcriptional activation of IL-1β- and TNF-α-induced matrix-degrading enzyme genes in chondrocytes. To this aim, human OA chondrocytes were pretreated with I-BET151, before exposure to IL-1β and TNF-α. Our results from a real-time PCR assay showed that *MMP1*, *MMP3*, *MMP13*, and *ADAMTS4* mRNA expression was robustly induced by IL-1β or TNF-α, and this induction was profoundly inhibited by I-BET151 (Fig. 2a–d). In response to IL-1β or TNF-α stimulation, a weak upregulation of *MMP2*, *MMP9*, and *ADAMTS5* gene expression was observed. I-BET151 repressed inducible transcription of *MMP2*, *MMP9*, and *ADAMTS5* (Fig. 2e–g). However, the repression of *MMP9* expression induced by TNF-α showed no statistical significance (Fig. 2f). Only *ADAMTS4* basal expression was significantly repressed by I-BET151 (Fig. 2a–g).

**Fig. 3:**
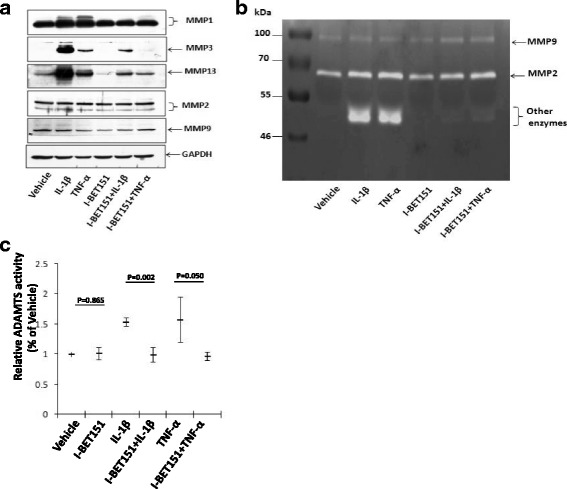
The effect of BET protein inhibitor on regulation of protein expression and activity of matrix degradation enzyme genes in human chondrocytes (**a**). The protein expression of MMP1, MMP3, MMP13, MMP2, and MMP9 proteins (Western blot) in human chondrocytes after the cells were pre-treated with or without I-BET151 (10 μM) followed by addition of IL-1β (10 ng/ml) or TNF-α (10 ng/ml) for 24 h (*n* = 1). **b** For MMP enzyme activity assay, gelatin zymography was performed using the cell culture supernatants that were obtained from culture human chondrocytes treated as in **a** (*n* = 1). **c** For ADAMTS4/5 enzyme activity assay, ELISA was carried out using the cell culture supernatants that were obtained from culture human chondrocytes treated as in **a** and enzyme activity was calculated by setting vehicle control activity to 100% (*n* = 3)

The results from Western blot showed that IL-1β and TNF-α strongly upregulated MMP1, MMP3, and MMP13 protein expression in human chondrocytes, and this upregulation was significantly reduced by I-BET151 (Fig. 3a). The protein expression of MMP2 and MMP9 was not significantly altered by IL-1β, TNF-α, and I-BET151, respectively (Fig. 3a).

### The activity of matrix-degrading enzyme is suppressed by BET protein inhibitor

We next examined the effect of I-BET151 on regulating matrix-degrading enzyme activity. We performed gelatin zymography assays using culture supernatants. Based on MMP molecular weight, the bands represented gelatinolytic activity of specific MMP. A strong induction of low molecular weight activity might indicate MMP1, MMP3, and MMP13, which was significantly reduced when the cells were pretreated with I-BET151. By contrast, the gelatinolytic activity of MMP2 and MMP9 was not significantly altered by IL-1β, TNF-α, and I-BET151, respectively (Fig. 3b). We next tested ADAMTS activity. To this end, culture supernatants were collected for aggrecanase activity analysis. I-BET151 abolished the IL-1β- or TNF-α-induced increase in the supernatant levels of enzyme activity (Fig. 3c).

### The expression of cartilage matrix genes and SOX9 in chondrocytes is suppressed by BET protein inhibitor

**Fig. 4:**
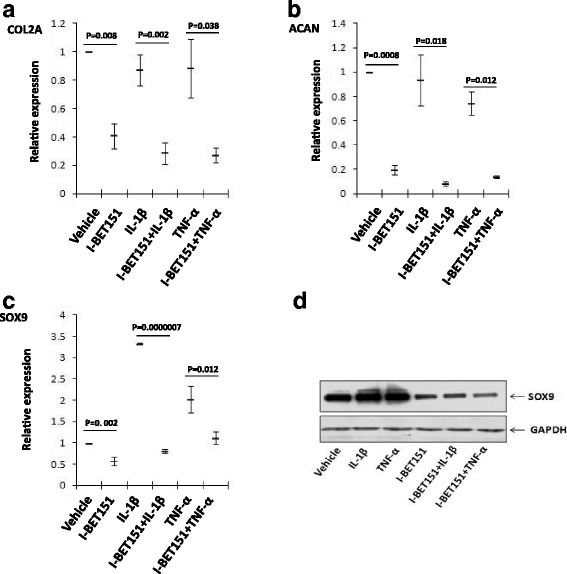
The effect of BET protein inhibitor on regulation of *ACAN*, *COL2A1*, and *SOX9* genes in human chondrocytes **(a**–**c**). The transcriptional expression (RT-PCR) of *ACAN*, *COL2A1*, and *SOX9* genes in human chondrocytes, respectively, after the cells were pre-treated with or without I-BET151 (1 μM) followed by addition of IL-1β (10 ng/ml) or TNF-α (10 ng/ml) for 24 h. Relative fold change values were calculated in comparison with vehicle control set as 1 (*n* = 3). **d** The protein expression of SOX9 proteins (Western blot) in human chondrocytes treated as in **a**–**c** (*n* = 1)

We investigated the roles of BET family proteins in the transcriptional activation of two cartilage matrix genes, aggrecan (*ACAN*) and collagen, type II, alpha 1 (*COL2A1*). The same RNA used above was applied for real-time PCR assay. The results showed that *ACAN* and *COL2A1* expression was inhibited by IL-1β or TNF-α, and this inhibition was significant aggravated by I-BET151 (Fig. 4a, b). We also investigated the expression of SRY-box 9 (*SOX9*) as SOX9 was an essential transcriptional factor for chondrocyte-specific genes [26]. And we found that I-BET151 could inhibit the expression of *SOX9* in human OA chondrocytes (Fig. 4c). The results of Western blot also showed that SOX9 was significantly inhibited by I-BET151 (Fig. 4d).

## Discussion

Consistent with previous reports [1–3], our results indicated that IL-1β and TNF-α upregulated the expression of several matrix-degrading enzyme genes including *MMP1*, *MMP2*, *MMP3*, *MMP9*, *MMP13*, *ADMATS4*, and *ADMATS5* in human chondrocytes. And the IL-1β- and TNF-α-induced expression of several matrix-degrading enzyme genes was significantly inhibited by I-BET151 in human chondrocytes, which was associated with the reduction of their enzyme activity. Our results indicated that BET proteins are required for IL-1β and TNF-α-induced transcription of those genes in chondrocytes but have weak effect on the basal expression of those genes in chondrocytes.

Our results showed that I-BET151 could repress the expression of *ACAN* and *COL2A1* genes in human chondrocytes when treated with IL-1β and TNF-α, and it also repress the basal expression of *ACAN* and *COL2A1* genes in chondrocytes. We also found that I-BET151 can repress the expression of *SOX9*. In a previous report, I-BET151 could repress the expression of Acan and Col2a1, but not of Sox9. It may because of different sources of the chondrocytes in two studies. The chondrocytes in our study were achieved from OA patients who underwent knee replacement surgery, and the chondrocytes in the previous study were achieved from newborn mouse [15]. Our result suggested that repression of *SOX9* contributed to the regulation of I-BET151 on the expression of matrix genes of chondrocytes, but other mechanisms might also occur in this procedure.

There have been reports that inhibiting IL-1β and inhibiting TNF-α can attenuate the OA progress of DMM mouse model by inhibiting inflammatory process, and the inflammation markers were enhanced in the synovium and chondrocytes of DMM mouse model [16, 17, 27]. In our study, we found that inhibiting BET proteins can rescue the degeneration of cartilage in the DMM mouse model at 6 weeks after treatment. The result was consistent with the previous reports as I-BET151 can suppress the IL-1β- and TNF-α-induced expression of cytokines and MMPs in both synovial fibroblasts and chondrocytes [14]. But we did not find significant effect of I-BET151 on the degeneration of cartilage in the DMM mouse model at 8 weeks after treatment. As we know, DMM was a surgical procedure which could cause joint instability, and inflammation should not be the cause for OA in DMM mouse model although there have been reports that inflammation was involved in the OA progress of this mouse model [16, 17, 27]. We suspected that effects other than inflammation might be the main etiology of OA in this mouse model, and these effects were not affected by I-BET151. It might be one reason for the attenuation of protecting effect at 8 weeks after treatment. We have found that inhibiting BET led to repression of *ACAN* and *COL2A1* in chondrocytes; it may be another reason for the attenuation of protecting effect at 8 weeks after treatment. The previous report stated that inhibiting BET can restrain bone growth, and there have been some reports indicated the importance of subchondral bone remodeling in etiology of OA [15, 28, 29], so the effect on subchondral bone by inhibiting BET might affect OA progress and needed further evaluation.

Some limitations should be noted in our study. First, we only analyzed the genes of interest in human chondrocytes which were treated in vitro but did not analyze the genes of interest in mouse which were treated in vivo. The expression of IL-1β and TNF-α was not evaluated in the mouse either. Although there have been reports that show inflammatory factors are involved in the OA progress of DMM mouse model and IL-1β is upregulated in synovium and chondrocytes of DMM mouse model [16, 17, 27], further research is needed to evaluate the inflammation-associated genes, including IL-1β and TNF-α, and the cartilage matrix genes at corresponding time (6 and 8 weeks after treatment) in the surgical joints of mouse which were treated in vivo. And it will be helpful to elucidate the reason for inconsistent in vivo results at different time. Secondly, we used a lower dose (10 mg/kg for in vivo treatment) of I-BET151 than the usual dose (30 mg/kg for in vivo treatment) in recent reports when we treated the surgical mouse [8, 13], although the dose (10 mg/kg for in vivo treatment) was mentioned in one previous report and showed anti-inflammatory effect [30]. The dose of reagent may affect the effect of treatment, and in vivo treatment with multiple doses is recommended for further study.

## Conclusions

In summary, we demonstrate that I-BET151 can protect articular cartilage from degeneration in a surgical mouse OA model at an early phase, but not a late phase. The reason of inconsistent in vivo results at different time needs further evaluation. I-BET151 can strongly inhibit inflammatory factor mediated upregulation of several matrix degradation enzymes including MMP1/2/3/13 and ADMATS4/5. On the other hand, I-BET151 was shown to significantly suppress the expression of *COL2A1* and *ACAN*. Thus, I-BET151 exerts a repression effect on both of chondrocyte anabolism and catabolism.

## Abbreviations

ADAMTS: A disintegrin-like and metalloproteinase with thrombospondin type 1 motifs
BET: Bromo and extra-terminal
DMM: Destabilization of the medial meniscus
DMSO: Dimethyl sulfoxide
GAPDH: Glyceraldehyde-3-phosphate dehydrogenase
IL: Interleukin
MMP: Matrix metalloproteinase
OA: Osteoarthritis
SOX9: SRY-related high mobility group-box gene9
TNF: Tumor necrosis factor
